# Restoring a post-traumatic partial edentulous mandible with the Toronto prosthesis: a clinical report

**DOI:** 10.15171/joddd.2018.021

**Published:** 2018-06-20

**Authors:** Fatemeh Nematollahi, Marzieh Alikhasi, Elaheh Beyabanaki

**Affiliations:** ^1^Department of Prosthodontics, Islamic Azad University, Dental Branch, Tehran, Iran; ^2^Dental Implant Research Center and Department of Prosthodontics, Tehran University of Medical Sciences, School of Dentistry, Tehran, Iran; ^3^Department of Prosthodontics, School of Dentistry, Shahid Beheshti University of Medical Sciences, Tehran, Iran

**Keywords:** Dental cement, dental implant, implant-supported dental prosthesis

## Abstract

Implants provide support, stability and retention for restorations used in fully and partially edentulous patients. This clinical report describes prosthetic treatment of a 30-year-old man suffering from a dentoalveolar deficiency due to an old gunshot trauma to the left side of the mandible. The patient was rehabilitated with implant-supported Toronto prosthesis following surgical placement of three implants with undesirable location and angulations due to lack of sufficient bone. This prosthetic option offers advantages of both screw-retained and cement-retained prostheses and provides acceptable aesthetic and functional results.

## Introduction

There are some critical factors for long-term success of dental implants, such as bone quality, position of implants^1-3^ and superstructure design in order to equally distribute occlusal loads among the implants.^4-6^ Although avoiding misaligned implants would help provide the patient with favorable esthetic and functional results, this is not always possible.^7,8^ There are some factors that inhibit augmentation of resorbed or defective alveolar ridges prior to implant placement, including surgical cost or patient health-related problems.^9,10^ Therefore it is expected that prosthetic restoration of mouth would also be complicated.

Generally there are two types of fixed implant-supported prostheses: screw-retained and cement-retained prostheses. Screw-retained restorations provide ease of retrievability of restoration for repair, hygiene measurements or retightening of abutment screw.^11^ However, this option may produce complications regarding location of screw access hole for misaligned implants, in terms of esthetic and occlusion.^12^ Moreover, if the metal framework design does not support the porcelain around the access hole, it might lead to porcelain fracture in this area.^13^

On the other hand, cement-retained restorations present several advantages over screw-retained restorations. These benefits include ease of fabrication, being aesthetically pleasing, simpler placement in posterior regions of the oral cavity and higher potential for achieving passive fit in the superstructure.^14,15^ However, they have some disadvantages such as difficulty in removing the restoration and remaining of cement excess around the implant.^16^

Toronto bridge technique^17^ or abutment-hybrid overdenture^18^ has been suggested in order to overcome problems of both types of aforementioned restorations and yet benefiting from their merits. In this technique the substructure is a screw-retained framework on which individual crowns would be cemented and pink or gingiva-colored porcelain or laboratory composite is used for mimicking the soft tissues.^16-20^ Rajan and Gunaseelan^21^ first described fabricating retrievable cement-retained and screw-retained implant prosthesis for single-tooth implant-supported restorations. This prosthesis offers the advantages of both cement-retained and screw-retained restorations such as retrievability of the abutment and the prosthesis, and easy removal of the prosthesis for cleaning the excess cement. The aim of this article was to present a case treated with Toronto prosthesis for restoration of partial edentulism in a post-traumatic mandible.

### Clinical Report

**Figure 1:**
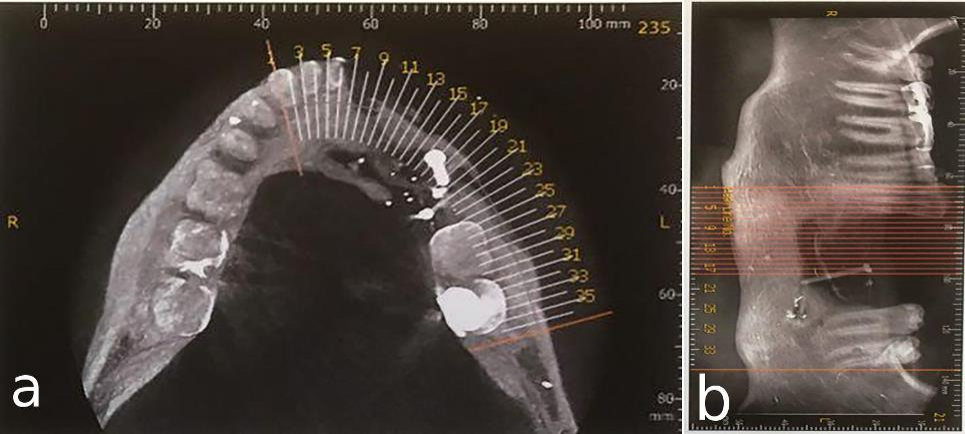
a) Occlusal view of the mandible after gunshot injury. b) Side view of the left side of the mandible after gunshot injury

**Figure 2:**
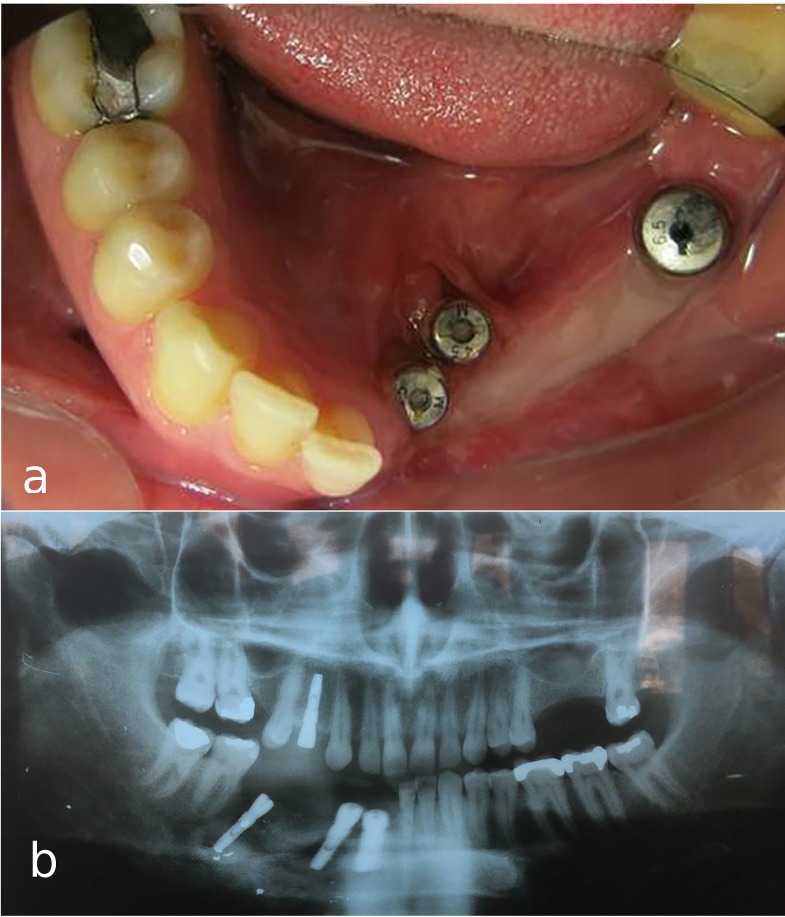
a) Occlusal view of mandibular arch with three implants. b) Panoramic view of implants placed in defective bone in the left mandible.

A 30-year-old partially edentulous patient was referred to the Implant Department of Tehran University, School of Dentistry, for prosthetic reconstruction (Figure 1, a and b). The patient had lost 6 teeth due to the trauma of a gunshot. Since the accident had occurred several years prevoiusly and the patient had undergone cosmetic plastic surgery, only a skin scar remained in his lower face. Three implants (Implantium, Dentium, Seoul, South Korea) were placed on the left side of the mandible (Figure 2, a and b). Regarding the increased interocclusal space and improper implant alignment, it was decided to use hybrid screw-retained and cement-retained implant (Toronto) prosthesis.

Using auto-polymerizing acrylic resin (Pattern Resin, GC, Tokyo, Japan) square impression copings were splinted, and an open-tray implant-level impression was made with polyvinyl siloxane impression material (Monopren Transfer, Kettenbach GmbH& Co., KG, Germany).^22^ Also, an irreversible hydrocolloid impression (Alginoplast; Heraeus Kulzer, Hanau, Germany) was made of the upper jaw. After attaching implant analogs to the impression copings in the impression, a silicone material (GI mask; Coltene/Whaledent, Cuyahoga Falls, Ohio) was injected around the coping analogs to serve as gingival tissues around the implants. Then, type IV dental stone (Hero Stone Vigodent Inc; Rio de Janeiro – Brazil) was used for pouring the impressions.

To record the jaw relationship, a screw-retained record base was made on the master cast. Before making the jaw relationship record, the occlusal surface of maxillary second premolar was reduced about 1 mm using a football-shaped diamond finishing bur (Tizkavan, Iran) to address its minimal supraeruption. After recording the centric position of the mandible with lower record base and occlusion rim opposing the upper teeth, using an inter-occlusal registration material (Virtual; IvoclarVivadent, Schaan, Liechtenstein), the casts were mounted in a semi-adjustable articulator (Dentatus ARH, Stockholm, Sweden) using a facebow transfer record. Then, acrylic denture teeth (Apple, Ideal Maco, Tehran, Iran) were set up on the record base and tried in the patient mouth for evaluation of tooth arrangement, position, length, gingival level and contour. Subsequently, a silicone (Panasil, Kettenbach GmbH& Co., KG, Germany) index was fabricated of the approved tooth set-up on the record base.

**Figure 3:**
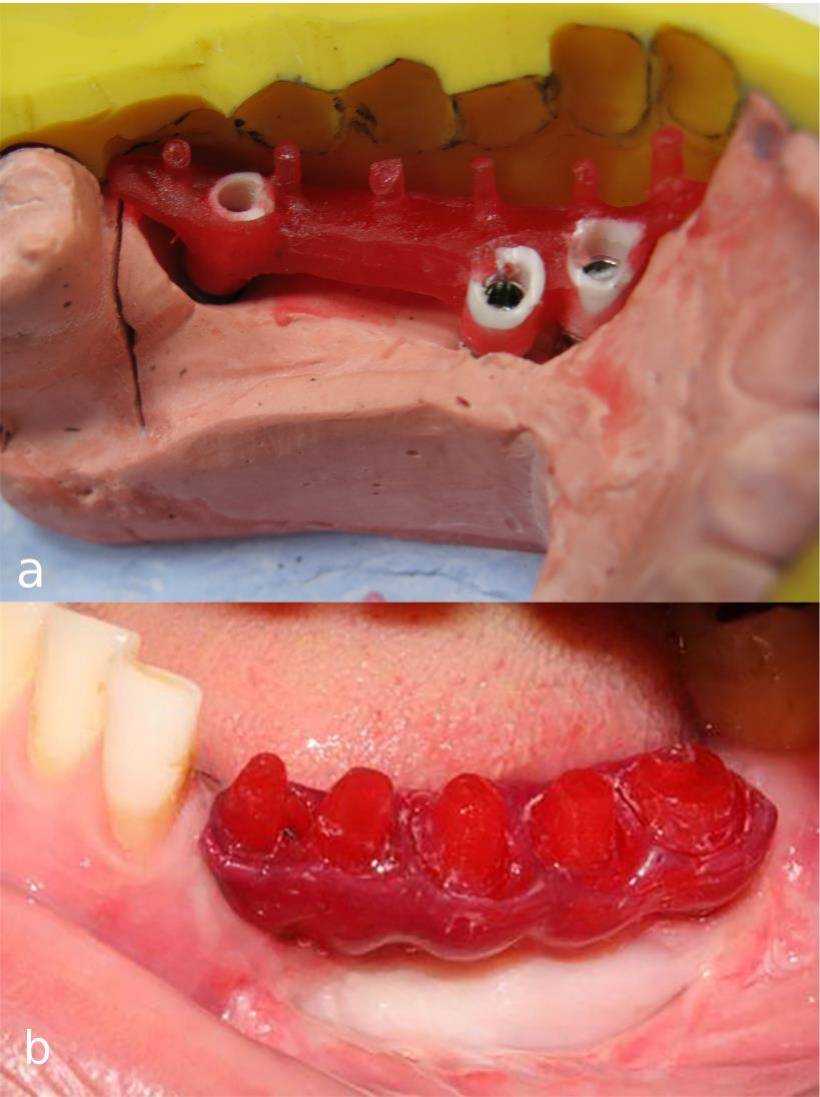
a) Fabrication of acrylic resin pattern of framework using plastic abutments. b) Try-in of the acrylic resin framework.

**Figure 4:**
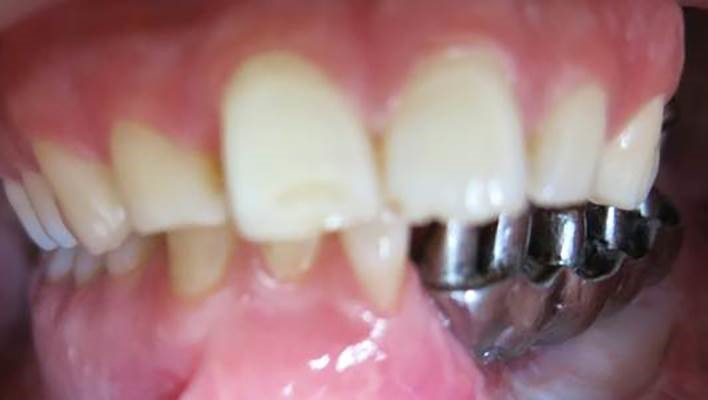
Try-in of the metal framework.

After connecting plastic abutments (burn-out sleeve) (Implantium, Dentium) to the implant analogs on the master cast with 10 N/cm torque, the acrylic resin (Pattern Resin, GC, Tokyo, Japan) pattern of infrastructure (meso-structure) was made using previously made silicone index as the guide (Figure 3, a). The pattern was then cut back in order to produce cores in the form of prepared teeth, and also to provide room for gingiva-colored porcelain replacing soft and hard tissue support. The resin pattern of the framework was tried in the oral cavity to verify the accuracy of impression procedure before casting (Figure 3, b). The passive fit of the cast framework with multiple individual abutments was confirmed in the oral cavity by means of one-screw test and periapical radiographs (Figure 4).^5,6^

**Figure 5:**
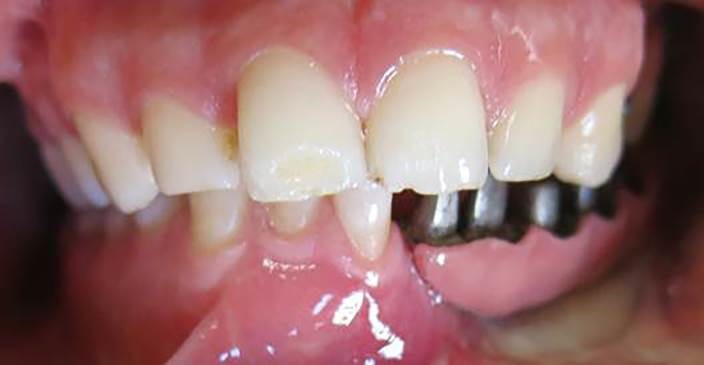
Try-in of metal framework with gingiva-colored porcelain.

Subsequently, gingiva-colored porcelain was baked on the gingival part of the framework by the aid of silicone index for controlling the gingival contour. After evaluating gingival adaptation of the framework intraorally (Figure 5), the copings of the superstructure (crowns) were directly waxed up on the framework. The fit of metal-ceramic crowns was assessed in the patient's oral cavity afterwards. Group function occlusion was created on the left working side to distribute functional loads on all the three implants.

**Figure 6:**
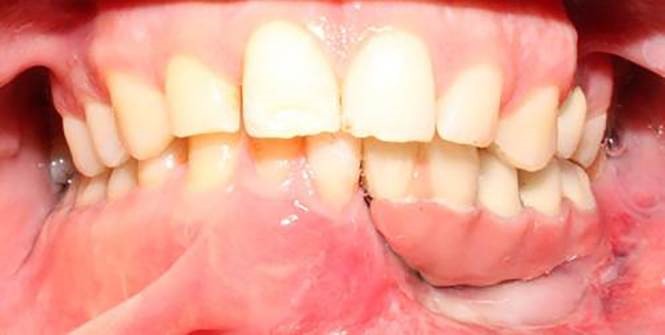
Delivery of prosthesis with cemented individual crowns.

Finally after screwing the meso-structure with 30 N/cm torque according to manufacturer’s instructions, Teflon tape (SITCO, Fujian China) was placed at the orifice of screw holes and covered with light-cured composite resin. Finally, the crowns were cemented with temporary cement (Temp-Bond, Kerr, Italy) on the metal framework (Figure 6).

## Discussion

The patient presented in this report was treated with a hybrid cement-retained and screw-retained implant restoration.‏ This kind of restoration is mainly indicated for demanding esthetic situations, excessive inter-occlusal space, and when vertical bone augmentation is not feasible.^16^ This kind of prosthesis could be used for restoring a single tooth,^21^ or partial^16,19^ and even a full^18,20^ edentulous arch. The main advantage of this kind of restoration is that not only implant position or angulation would not affect the design of the substructure, but also implant emergences could be corrected.^18^ Considering severe misalignment of the middle implant due to insufficient bone volume in the canine site as a result of an old gunshot injury in this case, this prosthetic option was selected. Also, regardless of location of screw access holes in the meso-structure, each crown can be fabricated in a position which is esthetically and functionally desirable.,^17^ Furthermore, when a great amount of porcelain is fired on the metal framework in cases of increased inter-occlusal space, distortion of the framework and loss of passive fit is expected. However, Toronto bridge technique prevents such distortion and also enables the possibility of replacing a damaged crown without jeopardizing the fit of the framework by re-firing it.^16-20^ Another advantage of this technique is that the esthetic and occlusion concerns usually seen in screw-retained restorations due to screw access hole are overcome.^17,18^ Moreover, retrievability of crowns cemented with temporary cement makes their maintenance relatively easy.^16-20^

However, the disadvantages entitled to this technique are difficulty of adjusting the crown's contact points, probability of crown dislodgement due to the use of a temporary cement, difficulty of achieving passive fit of screw-retained framework and high cost.^17-19^

This technique offers some varieties in terms of material used for framework, including casting,,^21^ zirconia^19^ and CAD-CAM milled titanium^20^ frameworks. Also, gingiva-colored composite resin^16^ could be used for covering the gingival part of the framework.

## Acknowledgment

None.

## Authors’ contributions

All authors contributed to the case selection, and treatment planning. FN, MA, and EB contributed to the treatment of the patient. FN and EB together drafted the manuscript. All authors have contributed to the critical revision of the manuscript, and have read and approved the final manuscript.

## Funding

Not applicable.

## Competing interests

The authors declare no competing interests with regards to the authorship and/or publication of this article.

## Ethics approval

The individual whose information is included in the report has given written informed consent for the publication of this paper.
